# Supplementary material for: Novel Driver Strength Index highlights important cancer genes in TCGA PanCanAtlas patients
Source: PeerJ. 2022 Aug 11;10:e13860. doi: 10.7717/peerj.13860 (PMC9375969; doi:10.7717/peerj.13860)
Supplement: Supplemental Information 2 [file peerj-10-13860-s002.zip › COHORTS/genes plots/detailed/2021_8_16_14_9_distribution_events_detailed_41.pdf]

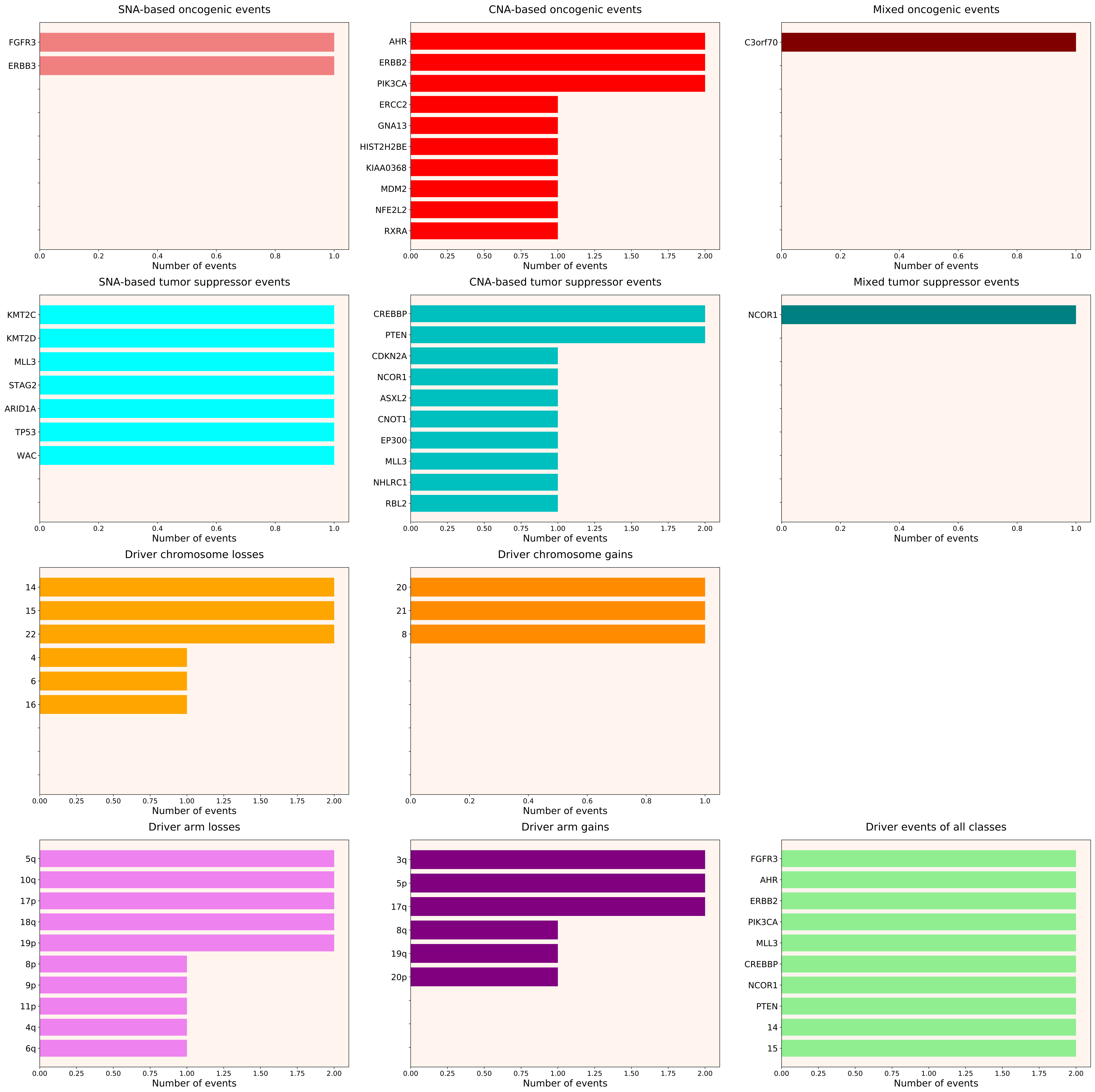
SNA-based oncogenic events
FGFR3
ERBB3
0.0 0.2 0.4 0.6 0.8 1.0
Number of events
CNA-based oncogenic events
AHR
ERBB2
PIK3CA
ERCC2
GNA13
HIST2H2BE
KIAA0368
MDM2
NFE2L2
RXRA
0.00 0.25 0.50 0.75 1.00 1.25 1.50 1.75 2.00
Number of events
Mixed oncogenic events
C3orf70
0.0 0.2 0.4 0.6 0.8 1.0
Number of events
SNA-based tumor suppressor events
KMT2C
KMT2D
MLL3
STAG2
ARID1A
TP53
WAC
0.0 0.2 0.4 0.6 0.8 1.0
Number of events
CNA-based tumor suppressor events
CREBBP
PTEN
CDKN2A
NCOR1
ASXL2
CNOT1
EP300
MLL3
NHLRC1
RBL2
0.00 0.25 0.50 0.75 1.00 1.25 1.50 1.75 2.00
Number of events
Mixed tumor suppressor events
NCOR1
0.0 0.2 0.4 0.6 0.8 1.0
Number of events
Driver chromosome losses
14
15
22
4
6
16
0.00 0.25 0.50 0.75 1.00 1.25 1.50 1.75 2.00
Number of events
Driver chromosome gains
20
21
8
0.0 0.2 0.4 0.6 0.8 1.0
Number of events
Driver arm losses
5q
10q
17p
18q
19p
8p
9p
11p
4q
6q
0.00 0.25 0.50 0.75 1.00 1.25 1.50 1.75 2.00
Number of events
Driver arm gains
3q
5p
17q
8q
19q
20p
0.00 0.25 0.50 0.75 1.00 1.25 1.50 1.75 2.00
Number of events
Driver events of all classes
FGFR3
AHR
ERBB2
PIK3CA
MLL3
CREBBP
NCOR1
PTEN
14
15
0.00 0.25 0.50 0.75 1.00 1.25 1.50 1.75 2.00
Number of events
